# Dilemma of HBsAg seroconversion in chronic hepatitis B infection: Dilemma of HBsAg in chronic HBV

**Published:** 2011-02-01

**Authors:** Seyed Moayed Alavian, Seyed Mohammad Miri

**Affiliations:** 1 Baqiyatallah Research Center for Gastroenterology and Liver Disease, Tehran, IR Iran

**Keywords:** Hepatitis B infection, HBsAg, Seroconversion, Occult hepatitis B, Iran

## Abstract

Patients with chronic hepatitis B infection should be followed up to identify possible changes in disease status, such as HBsAg seronversion. There are little data on the outcome of such cases, and the response rate to HBV vaccine has not been discussed extensively.

The prevalence of occult hepatitis B (OHB) infection in HBsAg-seroconverted patients has not yet been reported in the medical literatures. Due to the unknown outcomes of these patients who lost HBsAg and not seroconverted to anti-HBs with no detectable HBV DNA, Taheri et al. [1] determined the efficacy of HBV vaccine in such patients during 8 years of follow-up.Undoubtedly, this unique study opens a new window toward HBsAg seroclearnace. Their results strengthen the hypothesis that chronic HBsAg-positive cases who have lost HBsAg and are negative for serum HBV DNA in their sera may still have occult HBV infection [1]. Several factors influence the natural history of HBV infection, including the age at which the infection is acquired; viral characteristics, such as HBV genotype, viral mutations, and level of HBV replication, host factors, such as gender, age, and immune status; and exogenous factors, such as concomitant infection with other hepatotropic viruses or alcohol consumption. Prior to implementation of the national vaccination program for Iranian children, the main risk factor for acquiring HBV infection was perinatal infection from HBsAg-positive mothers to their infants during delivery [2][3][4]. Most patients with chronic HBV infection are inactive carriers, and there are an estimated 300 million inactive carriers worldwide. The diagnosis of inactive HBsAg carrier is based on the absence of HBeAg, the presence of anti-HBe, undetectable or low levels of HBV DNA in PCR-based assays, repeatedly normal ALT levels, minimal or no necroinflammation, slight fibrosis, and normal histology by biopsy [5]. According to published findings and due to variations in viral load, more than one HBV DNA test is required to make the diagnosis [6].

One of the principal outcomes of follow up of HBV inactive carriers is seronvesrsion which means becoming HBsAg negative and developing anti-HBs. There is a significant difference in HBsAg seroclearance rate between different studies and ethnicities. This difference could be owing to the difference in the occurrence time of HBV infection. In communities with more adolescent infections, such as western countries, the incidence of delayed HBsAg clearance ranges from 1% to 2% per year; while a lower rates, from 0.05% to 0.8% per year, are reported in endemic areas, where HBV infection is primarily acquired perinatally or in early childhood [7]. Although no Iranian report has been published, we estimate the incidence to be less than 0.5% per year (unpublished data).

With regard to the meaning of seroconversion, HBsAg seroclearance has been defined as the loss of serum HBsAg on 2 occasions at least 6 months apart, maintained to the study conclusion [8][9]. HBsAg seroclearance, which is associated with HBV DNA levels at baseline and follow-up, is one of the most important predictors of seroclearance. Higher HBV viral loads effect lower HBsAg seroclearance rates (P<0.001). A spontaneous decrease in follow-up HBV-DNA levels (>3 logs) was associated significantly with seroclearance, showed an adjusted odds ratio of 4.17 (95% confidence interval, 2.55-6.82). Among those who experience seroclearance, 95.8% have undetectable HBV DNA levels before seroclearance [10]. Further, the clearance of HBsAg has been reported to be higher in women than in men and in older carriers [7].Although the prognosis is improved by the loss of HBsAg-because the liver disease is usually inactive and nonprogressive-HBsAg clearance does not prevent the development of decompensation or Hepatocellular carcinoma in patients who have already become cirrhotic [11]. Occult hepatitis B (OBH) infection is an outcome of inactive carriers with HBV infection; thus, we should consider this issue in all cases of seroconversion, especially for patients in our region [12][13][14][15].According to the previous report about the effectiveness of HBV vaccine in anti HBcAb positive cases, we found that 20% of these cases had no respond to the vaccine. Among these patients, patients with a positive history of HBsAg were more likely to be unresponsive [16]. This finding supports the results of Taheri et al. Thanks to the main message of this article, however, limitations confessed by authors not to be ignored by readers. First, predictors, other than serum HBV-DNA and ALT levels, were measured only at baseline because some of these factors can change over time. Furthermore, they did not measure HBV viral load. Although age, ethnicity, and extreme obesity in men were significant predictors of HBsAg seroclearance, the comparison of serum HBV-DNA levels between baseline and follow-up might be the most important predictor of HBsAg seroclearance [10]. Also, the authors did not gather sufficient data regarding the exact time of HBV detection in their subjects [1]. And the third limitation is related to not forgetting one of the possible explanations of HBsAg negativity when there is low titer of HBsAg in the serum.
